# SpliceCenter: A suite of web-based bioinformatic applications for evaluating the impact of alternative splicing on RT-PCR, RNAi, microarray, and peptide-based studies

**DOI:** 10.1186/1471-2105-9-313

**Published:** 2008-07-18

**Authors:** Michael C Ryan, Barry R Zeeberg, Natasha J Caplen, James A Cleland, Ari B Kahn, Hongfang Liu, John N Weinstein

**Affiliations:** 1 Genomics & Bioinformatics Group, Laboratory of Molecular Pharmacology, National Cancer Institute, National Institutes of Health, Bethesda, Maryland, USA; 2 Tiger Team Consulting, Fairfax, VA, USA; 3 Gene Silencing Section, Genetics Branch, National Cancer Institute, National Institutes of Health, Bethesda, Maryland, USA; 4 Department of Biostatistics, Bioinformatics, and Biomathematics, Georgetown University Medical Center, Washington, DC, USA; 5 Department of Bioinformatics and Computational Biology, M. D. Anderson Cancer Center, Houston, TX, USA

## Abstract

**Background:**

Over 60% of protein-coding genes in vertebrates express mRNAs that undergo alternative splicing. The resulting collection of transcript isoforms poses significant challenges for contemporary biological assays. For example, RT-PCR validation of gene expression microarray results may be unsuccessful if the two technologies target different splice variants. Effective use of sequence-based technologies requires knowledge of the specific splice variant(s) that are targeted. In addition, the critical roles of alternative splice forms in biological function and in disease suggest that assay results may be more informative if analyzed in the context of the targeted splice variant.

**Results:**

A number of contemporary technologies are used for analyzing transcripts or proteins. To enable investigation of the impact of splice variation on the interpretation of data derived from those technologies, we have developed SpliceCenter. SpliceCenter is a suite of user-friendly, web-based applications that includes programs for analysis of RT-PCR primer/probe sets, effectors of RNAi, microarrays, and protein-targeting technologies. Both interactive and high-throughput implementations of the tools are provided. The interactive versions of SpliceCenter tools provide visualizations of a gene's alternative transcripts and probe target positions, enabling the user to identify which splice variants are or are not targeted. The high-throughput batch versions accept user query files and provide results in tabular form. When, for example, we used SpliceCenter's batch siRNA-Check to process the Cancer Genome Anatomy Project's large-scale shRNA library, we found that only 59% of the 50,766 shRNAs in the library target all known splice variants of the target gene, 32% target some but not all, and 9% do not target any currently annotated transcript.

**Conclusion:**

SpliceCenter  provides unique, user-friendly applications for assessing the impact of transcript variation on the design and interpretation of RT-PCR, RNAi, gene expression microarrays, antibody-based detection, and mass spectrometry proteomics. The tools are intended for use by bench biologists as well as bioinformaticists.

## Background

Technologies commonly used by biologists to investigate gene function include quantitative RT-PCR (qRT-PCR) assays, RNA interference (RNAi) mediated by small interfering RNAs (siRNAs) or short hairpin RNAs (shRNAs), gene expression microarrays, and antibody-based protein assays. Each of those technologies targets a small nucleic or amino acid sequence that, preferably, is unique to a specific gene.

More than 60% of protein-coding genes in vertebrates exhibit splice variation [1,2]. Alternative splicing complicates the selection of target sequence and interpretation of resulting data. In many cases, the targeted sequence might not be present in all of a gene's transcript forms. The prevalence of alternative spliceforms suggests many questions that routinely confront biologists who use oligonucleotide- or peptide-based assays. For example:

• Which specific splice variants are targeted by my assay? What other splice variants exist?

• Do the RT-PCR primers/probes that I plan to use to validate microarray expression results target the same splice variants as were targeted by the microarray platform?

• Did an siRNA fail to mediate RNAi silencing of a gene because it did not target the dominant splice isoform?

• Is there known splice variation in my gene of interest that affects the protein coding portion of the transcript? Does the antibody that I plan to use target all potential protein products?

• Where could I place RT PCR primers to target all splice variants? Where could I place RT PCR primers to amplify one specific splice variant to the exclusion of others?

• Do expression values from one microarray fail to correlate with values from another microarray because the probesets target different splice variants?

Questions such as those are not motivated by a particular research focus on alternative splicing, but rather by the need to account for the impact of splice variation in almost every high-throughput biological study. In our laboratories, for example, we have experienced several such issues, including an siRNA that failed to target the dominant transcript in a particular cell line and RT PCR results that failed to correlate with microarray expression data because different splice forms were targeted [3].

In addition to the pragmatic motivations for evaluating the splice variants targeted by a given assay, there may also be scientific benefit. Alternate splice forms have been associated with tissue-specific gene functions, developmental processes, and disease states (notably cancer) [4,5]. Genes with splice variation in the coding region produce different proteins with potentially dramatically different functions. For example, the Epidermal Growth Factor Receptor (*EGFr*), a major target for cancer therapy, can be expressed in the transmembrane receptor form or as a soluble isoform that competes with the receptor for binding of ligand. Transcripts with variation in the untranslated regions (UTRs) may be differentially regulated and therefore exhibit differences in spatial or temporal expression patterns.

Several publicly-accessible websites provide data and utilities for investigating alternative splicing: AceView [6], Alternative Splicing Annotation Project database (ASAP II) [7], Alternative Splicing and Transcript Diversity Database (ATSD) [8], Friendly Alternative Splicing and Transcript Database (fastdb2) [9], Hollywood [10], Eukaryotic Splice Database (EUSplice) [11], and Genome Annotation for Alternative Splicing (ECgene) [12], and Splicy [13]. Each of those databases provides information on transcript variants and in some cases additional annotation, including splicing regulatory elements, exon characteristics (*e.g*. alternative, constitutive, retained, internal), and EST evidence/tissue of origin data. Although some of those programs are excellent resources for alternative splicing data, none of them provide query features and output focused on, and tuned to, the need of bench biologists to understand the impact of alternative splicing on their sequence-based or hybridization-based assays. A comparison of SpliceCenter with the other available resources is summarized in Table 1.

**Table 1:** Comparison of SpliceCenter features with those of other web-based splicing resources

**SpliceCenter Capability**	**AceView**	**ASAP**	**ATSD**	**fastdb2**	**Hollywood**	**EUSplice**	**ECgene**	**Splicy**
General								
Simple, Intuitive Interface						✔		
Help and Sample Queries	✔	✔	*	*	*	✔	✔	*
Splice Variants								
Graphical display of gene's splice variants	✔	✔	✔	✔	✔	✔	✔	✔
Identifies coding regions	✔		✔			✔		
Identifies NMD targets				✔				
Human, Mouse, and Rat	*	✔	✔	*	*	✔	✔	*
PCR								
Primer sequence query showing position in variants				✔			✔	
Batch high-throughput primer query								
siRNA								
siRNA sequence query showing position in variants								
Batch high-throughput hit/miss report for siRNAs								
Microarray								
Display pre-computed target positions of Affymetrix, Agilent, Illumina, and ExonHit probesets in gene's variants								*
Display integrated graphic of microarray probe targets and primer target positions.								
Batch high-throughput query of pre-computed probe/probeset target positions								*
Peptide								
Peptide sequence query displaying source coding region in splice variants								
Batch high-throughput peptide query								

This table shows the capabilities of SpliceCenter in the first column and identifies other splicing database websites with similar features. The comparison is done from the point of view of a biologist interested in investigating the impact of alternative splicing on a sequence-based assay. Other sites contain features not found in SpliceCenter but which are not directly applicable to the focused tasks addressed by our tools. A '✔'indicates presence of a feature, a blank cell indicates absence of a feature, and a '*' indicates partial implementation of a feature.

To meet the needs of biologists as well as bioinformaticists, we have developed SpliceCenter, a comprehensive, rapid, user-friendly suite of web-based tools for identifying the alternative transcripts targeted by contemporary technologies. The SpliceCenter tools include: "Primer-Check" for evaluation of qRT-PCR primers/probes, siRNA-Check" for analysis of RNAi effectors, "Array-Check" for analysis of mRNA expression microarray probes, and "Peptide-Check" for analysis of peptides (*e.g*., in antibody-based binding assays and mass spectrometry). SpliceCenter applications provide the novel ability to cross-compare the technologies (*e.g*., with a single graphical visualization that indicates both the splice forms of a gene that are targeted by RT-PCR primers and those that are targeted by a microarray probe set).

SpliceCenter was developed using infrastructure from our previously described SpliceMiner application [14] including the splice variant database structure and microarray probe assignment features. SpliceCenter represents a substantial advance over our previous work in terms of the database content, website utilities, and breadth of potential users. The database was rebuilt and extended to include the latest genomic and transcript data, mouse and rat genomes, position of coding regions, identification of NMD targets, protein sequences, and pre-computed microarray probe targets. The new website utilities provide custom, user-friendly applications tailored to the needs of bench biologists who are applying such technologies as RT-PCR, RNAi, microarrays, or peptide-based assays. The focused nature of SpliceCenter utilities provide a significant time savings compared to our previous application [see Additional file 1]. Additional query facilities were also implemented to process protein sequences and very short oligos (*e.g*. siRNA and PCR primer sequences).

## Construction and Content

**Figure 1:**
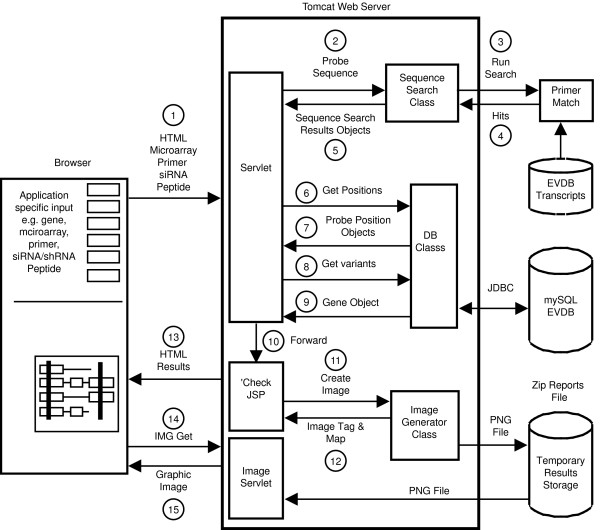
**A component interaction diagram of the SpliceCenter software.** The processes depicted in the figure are keyed to the following steps: (1) The HTML Browser submits a form that contains the user query specifying a gene and a microarray, PCR primer sequences, siRNA sequences, and/or peptide sequences. (2) The HTML request is received by a utility-specific servlet that performs input validation. The servlet calls the Sequence Search class providing the primer, siRNA, or peptide sequence as input. (3) The Sequence Search class runs the open source sequence search tool PrimerMatch [16] to locate complementary sequences within a database of unique splice variant transcripts. (3) PrimerMatch returns hits for the query sequences. The Sequence Search class parses the PrimerMatch results and identifies the best match. (4) Sequence Search Result objects are returned to the servlet. (5) Transcript coordinates for alignment of the hits are converted into genomic coordinates by querying EVDB. Also, the user-specified microarray and gene symbol are used to identify microarray probe positions for the specified gene. (6) Genomic coordinates for each query sequence are packaged into position objects. Microarray probe coordinates are returned in probe-position objects. (7) The user-provided gene symbol (or automatically derived target gene for siRNAs or peptides) is used to query all known splice variants in EVDB. (8) Gene, Variant, and Exon objects are returned. Those objects provide the genomic coordinates of the exons in each unique splice variant. (9) Gene objects and position objects are forwarded in the HTML Request to the PrimerCheck Java Server Page. The JSP is responsible for presentation of search results. (10) The JSP calls the ImageGenerator class, which uses the gene and position objects to construct a graphical image of the splice variants and target positions. To create a graphical output, the ImageGenerator class uses the Java AWT classes, including Graphics2D. Graphical images are temporarily stored on the disk drive on the Tomcat Server. (11) An image tag, complete with a tooltip map, is returned to the JSP for inclusion in the results page. (12) An HTML page with the search results is returned to the user's browser. (13) The image tag on the results page refers to the Image Servlet with a unique ID of the user's result image. The browser automatically requests the image from the Image Servlet. (14) The Image Servlet delivers the PNG format image file and cleans the image file from temporary storage.

SpliceCenter applications are web-based Java programs (JSPs and servlets) that query two mysql databases: Evidence Viewer Database (EVDB) and Microarray Database. EVDB contains the complete coding transcripts from RefSeq(release 29) and GenBank(release 165) that represent distinct splice variants. (See reference [14,15] for details on the contents and construction of EVDB). The Microarray database contains pre-computed probe target positions for more than 11 million probes covering 32 commercial microarrays [see Additional file 1]. Figure 1 provides a high-level overview of the software architecture, which follows a traditional model-view-controller pattern.

We needed to develop new sequence-alignment components for PCR-Check and siRNA-Check because the relatively short sequence lengths (*e.g*., 21 nucleotide sequences of siRNAs) were problematic for our previously developed sequence alignment functions. The new sequence search component makes use of the open source PrimerMatch [16] application to align user-provided PCR primer or siRNA sequences with transcript sequences. We selected PrimerMatch because, unlike BLAT and BLAST, it can rapidly and accurately align very short query sequences.

For Peptide-Check, a peptide-to-protein alignment is performed with the translated protein sequences from the RefSeq and GenBank records in our splice variant database. Alignments are converted into chromosomal coordinates, and thus into the transcript position, *via *EVDB mappings.

**Figure 2:**
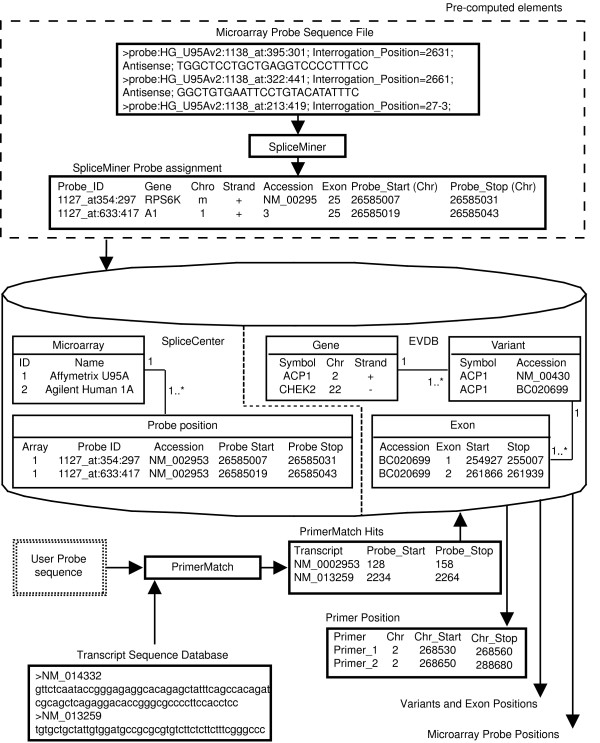
**An overview of the data processing performed to determine the positions targeted by microarray probes, PCR primers, peptides, or siRNA/shRNAs and to retrieve/display the exon structures of splice variants.** The data processing flow in SpliceCenter is as follows: (1) SpliceMiner is used to pre-compute target positions for common commercial microarray probes. Microarray probe sequence files are submitted to the batch query facility of SpliceMiner, and a results file then indicates the target of each probe in genomic coordinates. The results are loaded into the Probe Position and Microarray table of the mySQL database used by SpliceCenter. (2) EVDB data consisting of a list of the unique splice variants for each human gene are loaded into the SpliceCenter database. Genomic coordinates for each exon are specified by those data. (3) For analysis of RT-PCR, PrimerMatch searches a transcript sequence database to locate the primer target positions that correspond to the PCR primer sequences. PrimerMatch results indicate the target positions for PCR primers in transcript coordinates. (4) The Gene/Variant/Exon tables in the database are queried using the transcript coordinates of the PCR primer. The database results are used to convert PCR primer targets into genomic coordinates. (5) A complete list of the gene's splice variant exon positions and pre-computed microarray probe positions is retrieved from the database. (6) The genomic position information for splice variant exons, microarray probes, and PCR primers is passed to the ImageGenerator to construct the graphical image results.

An overview of the strategy for determining the positions targeted by PCR primers or siRNA/shRNAs is provided in Figure 2. A key processing step performed by SpliceCenter applications is conversion of various elements into a common frame of reference, genomic coordinates. The target positions of user-supplied sequences and microarray probes are determined by a 100% identity match to transcript or protein sequences in our database. Identifying the genomic coordinate positions of splice variants, exons, microarray probes, PCR primers, siRNAs, shRNAs and peptides allows the creation of a graphical image that displays each of those elements accurately and consistently.

## Utility and Discussion

The SpliceCenter web-based application suite is designed to assist biologists and bioinformaticists in analyzing the impact of alternative splicing on studies of transcripts and proteins. Each application identifies the target locations of oligonucleotides or peptides within the unique splice variants of the targeted gene or genes. Interactive investigation is supported through web-based applications that return graphical results with clickable hyperlinks. High-throughput applications for processing large query files are implemented as batch applications that return text-based result files. Currently support species are human, mouse, and rat.

### Primer-Check

**Figure 3:**
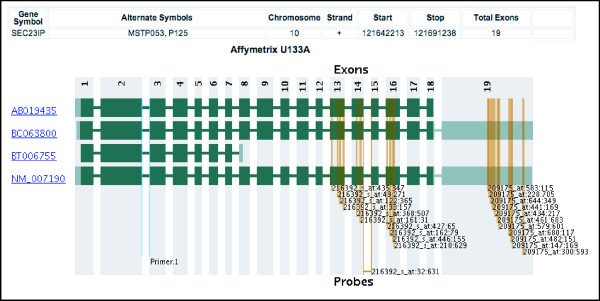
**Graphical output of Primer-Check for *SEC23IP *(P125) showing the discrepancy between variants targeted by the Affymetrix U133A array probe sets and Taqman (ABI) primer and probe sets**[24]**used by Dallas and co-workers**[17]. Four distinct splice variants of *SEC23IP *are displayed. Orange vertical lines indicate the target positions of the microarray probes. Probe set 209175_at targets two of four variants, and probe set 216392_at targets three of four variants. The light blue vertical lines show the context sequence location for the qRT-PCR ABI Taqman primer/probe set that targets all splice variants. The dissimilarity of splice variant coverage between qRT-PCR primers and microarray probes creates the potential for discordant expression values, depending on which mix of variants is expressed in the sample being studied.

Quantitative RT-PCR (qRT-PCR) is widely accepted as the gold standard for validation of microarray expression studies and is also widely used in its own right for accurate measurement of mRNA levels. However, the process of designing oligonucleotide primers for qRT-PCR requires that all existing transcript variants be considered. For example, if qRT-PCR primers and microarray probes target different splice variants, validation of expression results may be compromised. Primer-Check shows the splice variants of a gene and indicates the target locations of PCR primers specified by the user, thereby allowing rapid determination of which variants are and are not targeted. That information can help in the design or selection of primers or in diagnosing unexpected results. The Primer-Check application also includes the option to construct graphical results that indicate the positions of both PCR primers and microarray probes to evaluate whether the two target the same variants. The user enters the target gene and sequence of the oligonucleotides selected for use as primers and/or detection probes. Primer-Check returns a graphical display of the splice variants of the gene and indicates the target locations of the primers (Figure 3). Common usage scenarios include the following:

• Primer design and selection: Whether designing custom primers or selecting commercial ones, it is important to identify the splice variants that will be targeted. Primer-Check can be used to ensure that selected primer pairs hybridize to all variants (or specifically targeted variants) and to screen for possible cross-hybridizations.

• Investigation of anomalous results: One potential reason for failure of RT-PCR primers is that they are not targeting the splice variant(s) present in the particular sample being analyzed. Primer-Check is useful for trouble-shooting RT-PCR primers that fail to provide the expected amplification product.

• Validation of microarray data: As already noted, qRT-PCR is considered to be the gold standard for validation of microarray results. Primer-Check can display the target locations of PCR primers and probes and the target locations of microarray probes in a single graphical display that shows directly whether PCR primers and microarray probes do, in fact, target the same variants.

The effect of splice variation on validation of microarray expression data by qRT-PCR data is by no means hypothetical. A study by Dallas and colleagues [17] found that such correlations were negatively impacted by splice variation if PCR primers and microarray probes targeted differently spliced transcripts. Figure 3 shows Primer-Check results for *SEC23IP *(*P125*), a gene that showed discordance between microarray and qRT-PCR results in the Dallas study [17]. Primer-Check identifies four known splice forms of the gene. Two Affymetrix probe sets on the U133A GeneChip target the RefSeq-annotated transcript (NM_007190) that corresponds to SEC23IP. However, one of those probes sets, *216392_s_at*, also targets two additional transcript variants (BC063800 and AB019435), missing the fourth. The second probe set, *209175_at*, targets only one of those additional variants (BC063800), missing the other two. In contrast, the PCR primers and probes from Applied Biosystems target all four reported variants corresponding to *SEC23IP*, leading to discordance between the qRT-PCR and microarray results. Primer-Check can help diagnose or even avoid such problems.

### siRNA-Check

RNAi technologies based on exogenously administered siRNAs or shRNAs are used extensively to investigate gene function. For compactness in the following descriptions, we will often use the term "siRNA" to include all of the standard RNAi effector molecules. siRNAs mediate sequence-specific gene silencing through targeted cleavage of a transcript *via *the RNA interference pathway. Selecting an siRNA sequence that effectively targets a gene is a complex task that requires *in silico *prediction of the ability of the siRNA to mediate cleavage of the targeted transcript(s) while avoiding partially homologous sequences of other genes. Databases of experimentally-validated siRNAs and several tools to aid in design are available [18,19]. To achieve the goal of maximally silencing protein expression, it is safest to ensure that all protein-encoding transcript variants are targeted by the siRNA. Hence, in most cases siRNAs have been designed to target all splice variants of a gene that are found in the RefSeq database. But because RefSeq was not designed or intended to include all known transcripts, non-RefSeq splice variants may not be targeted. If, for example, an siRNA has been successful in one cell type and then fails to silence expression in another, the two cell types may be expressing different splice variants. siRNA-Check can be used to confirm targeting of all known variants or selective targeting of a particular variant (and, therefore, silencing of a particular protein isoform). The following are typical uses of siRNA-Check:

• Selection or design of siRNA (or shRNA) sequences: Whether designing custom siRNAs or selecting commercial ones, it is important to understand which variants will be targeted. The siRNA-Check application can be used to confirm targeting of all variants or selective targeting of a particular variant (and, therefore, silencing of a particular protein isoform). In interactive mode, the application identifies siRNA target sequences within a gene *via *an intuitive graphical display. If an siRNA targets a sequence that occurs in more than one gene, multiple graphics panels, one for each gene, are displayed.

• Clarification of anomalous results: siRNA-Check provides a quick, easy way to investigate the possibility that failure to silence a gene is due to splice variation. To cite one example from our own work, when we were trying to knock down expression of two apoptosis-associated genes, *BAD *and *YWHAZ*, we observed differential expression of some of the untargeted transcript variants [3]. For example, as shown in Additional Figure 1a [see Additional file 2], two siRNAs that target *BAD *(siBAD.1 and siBAD.3) mediated a significant decrease in mRNA levels when all variants of the gene were assayed (using the Branched DNA-RNA Quantigene assay, Panomics, Fremont, CA). But siBAD.2 produced no knockdown. Transcript-specific qRT-PCR showed that NM_004322, the transcript variant targeted by siBAD.2, represents only 1% of *BAD *mRNA levels in the cell line studied. We saw analogous results for the gene *YWHAZ *(Additional Figure 1b) [see Additional file 2].

### Array-Check

**Figure 4:**
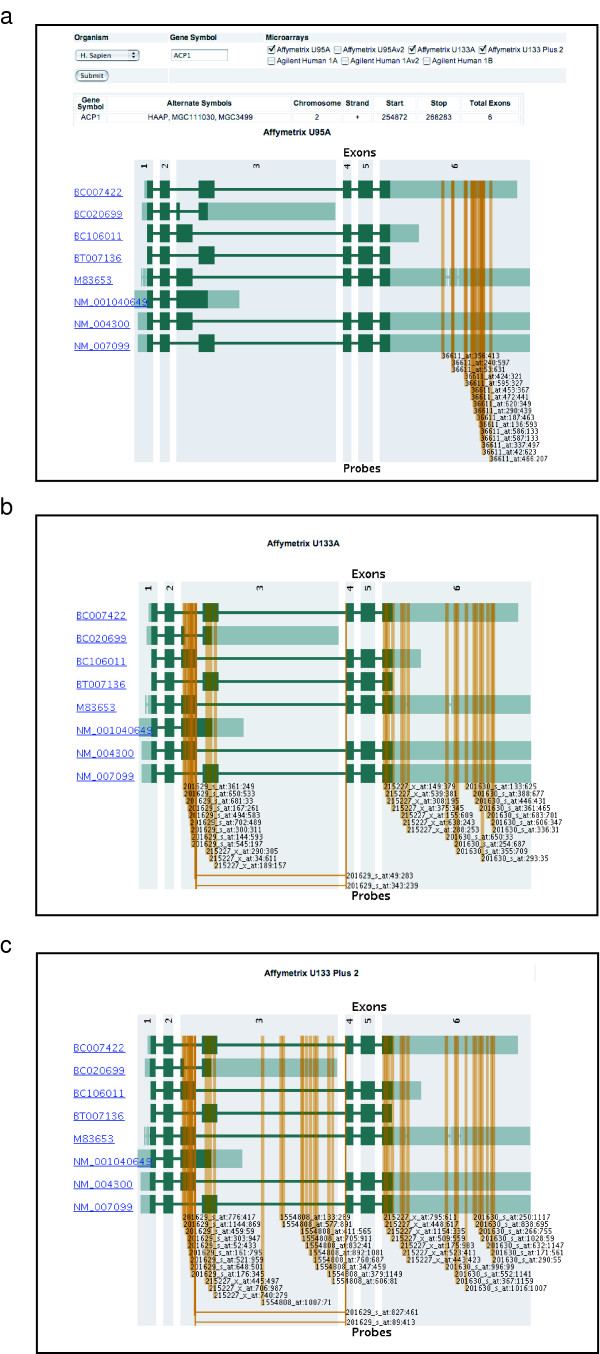
**Graphical output of Array-Check showing the known splice variants of *ACP1 *and the positions of oligonucleotides used to target the gene on (a) Affymetrix U95A, (b) Affymetrix U133A, and (c) Affymetrix U133 Plus 2 microarrays.** The splice variants are identified by accession number. Green boxes, which indicate exons, are drawn to scale. Lighter green regions of the exons are UTRs, and dark green areas are the coding regions. Thin connecting lines, which indicate introns, are not to scale. Orange vertical lines show the target locations of microarray probes. Probe lines intersecting exons indicate transcripts detected by the probe. Horizontal orange tie lines indicate splice junction probes (*i.e*., probes that cross exon boundaries).

Transcript expression microarrays are being used as tools throughout basic and clinical research. The results of microarray expression studies are usually reported as lists of over- or under-expressed genes. However, failure of the oligonucleotide probes to detect all splice variants of the target gene may confound interpretation of the results. For example, a recent analysis of gene expression data obtained for different microarray platforms showed that genes exhibiting transcript variation had a lower signal agreement between platforms than did genes with less alternative splicing [20]. Array-Check enables the user to see at a glance which variants are targeted by a given microarray platform and, if desired, to compare the variants targeted by different microarray platforms (Figure 4). Array-Check includes a database of pre-computed microarray probe target data for the most widely-used commercial expression microarrays. The splice variant coverage of a microarray should be taken into account in a variety of research situations. The following are examples:

• Microarray platform evaluation: Figure 4 shows an Array-Check comparison of Affymetrix 95A, U133A, and U133 Plus 2 in their coverage of the *ACP1 *gene. Array-Check thus provides a quick means of performing a side-by-side comparison of the coverage of splice variants by microarray platforms. It can be used in the mining of historical microarray datasets to ensure that an older platform provided good coverage of all variants of the gene of interest. It may also be useful in selecting a platform for a new study. As shown in the figure, the U133A and U133 Plus 2 Affymetrix arrays provide better coverage of the *ACP1 *variants than does the U95A array.

• Microarray platform correlation: Comparison of expression values from different microarray platforms is prone to misinterpretation if the platforms target different splice variants. Array-Check provides a mechanism for comparison of probe target locations to identify potential splicing-related differences. For example, Figure 4 shows that correlation between probe set 36611_at on the U95A array and probe set 1554808_at on the U133 Plus 2 array is unlikely because those probe sets measure non-overlapping subsets of the splice variants of *ACP1*.

• Trouble-shooting anomalous results: Alternative splicing is a potential source of inconsistent expression measurements among the probes in a nominal probe set. Array-Check provides a rapid means for ascertaining the known variants that are targeted or missed by probes on a given microarray platform. Older microarrays were designed before the availability of detailed annotation of many of the recently-identified transcript variants. Array-Check indicates splice variant coverage in the context of up-to-date information on transcript variation.

### Peptide-Check

Alternative splicing plays a critical role in higher organisms by increasing the functional diversity of proteins. Isoforms that differ minimally in structure may perform very different functions or may perform the same function in different cell types or at different stages of development. Failure to take splice variation into account can lead to inaccurate or incorrect interpretation of experimental results. Mass spectrometry and antibody-binding assays, the most common technologies in proteomic research, are susceptible to such problems. For those technologies, Peptide-Check provides a simple interface that accepts one or more short peptide sequences, generates a visualization of the known splice variants of the source gene, and shows the location, within the mRNA transcript, of the nucleotide sequence that codes for the peptide. Common use-cases include the following:

• Design and analysis of peptide immunogens and antigens: Peptide-Check has many applications in the context of technologies that use antibodies or other ligands that target peptide sequences. To cite one common example, animals are often immunized with a peptide to generate antibodies against a specific protein. Peptide-Check can assist in selecting an immunizing peptide that occurs in all splice forms of the protein or, conversely, in only one particular form. The latter type of specificity may be particularly useful for identification of the biological or pathological roles of individual protein isoforms. For example, antibodies raised against peptides that represent unique splice variants of p53 have helped to elucidate details of the molecule's tumor suppressor function [21]. Peptide-Check provides a rapid method for identifying the target variants of those p53 antibodies [see Additional file 3]. It should be noted that Peptide-Check is capable of processing only sequential peptide epitopes; it cannot help with conformational epitopes that are composed of multiple sequences within a protein.

• Analysis of Mass spectrometry results: Mass spectrometry is increasingly being used to identify and/or quantify proteins in a biological sample after peptidolysis. The first step is to identify peptides on the basis of mass/charge ratios, partial sequences, and/or chromatographic elution times. The identity of the original protein is then inferred from the peptides by any of a number of available software packages (reviewed in [22]). Peptide-Check can then be queried to explain the presence or absence of peptides that correspond to a given protein isoform and perhaps to give information on which isoforms are expressed in the sample. In principle, knowledge of splice variation could also be included in calculation of the protein identification probabilities provided by peptide fingerprinting programs.

### Batch Utilities

**Figure 5:**
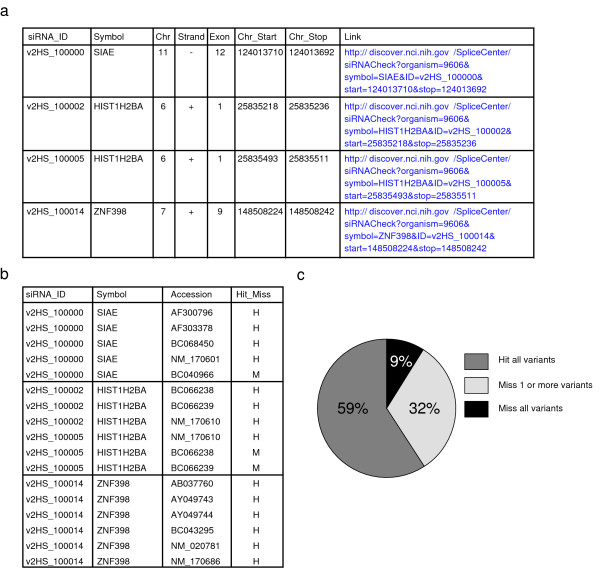
**Output of batch siRNA-Check used for high-throughput analysis of siRNA or shRNA libraries.** (a) The siRNA Position Report indicates the target location of each siRNA sequence, including gene symbol, chromosomal coordinates, and a link to a graphical view of the target gene. (b) The Hit/Miss report indicates which transcripts will, and which will not, be targeted by each siRNA sequence. (c) Summary statistics from a batch siRNA-Check analysis of the unique shRNA sequences of a 50,000+ shRNA library [23]. The chart indicates the percentage of shRNAs that do not target any currently annotated transcript, that target some but not all transcripts of the target gene, and that target all known splice variants of the target gene.

Each of the interactive SpliceCenter tools has a corresponding batch application for high-throughput processing of query files. For example, the batch form of siRNA-Check accepts a FASTA-format file containing multiple RNAi effector sequences (*e.g*. siRNA or shRNA). Two result files are produced: 1) the Hit/Miss Report and 2) the siRNA Position Report (Figures 5a and 5b). For each query sequence, the Hit/Miss Report indicates the gene(s) targeted by the siRNA sequence as well as the splice variants that are, or are not, targeted. The siRNA Position Report provides the genes, exons, and chromosomal coordinates targeted by each query sequence. Batch siRNA-Check can be used to evaluate large libraries of siRNAs or shRNAs. The Hit/Miss report is useful for identifying those that fail to silence all known variants of a gene or those that target just a single transcript of interest. For detailed evaluation of a specific entry, the Position Report provides a hyperlink to an interactive-mode graphical representation of the splice variants and positions of the siRNA sequences. To illustrate the use of batch siRNA-Check, we processed the publicly available sequences of a large-scale shRNA library (constructed by Hannon and co-workers) available as part of the Cancer Genome Anatomy Project (CGAP) [23]. The reports produced by batch siRNA-Check identified the targets of each shRNA (Figure 5c). Of the 50,766 shRNAs in the library, 59% target all known splice variants of the target gene, 32% target some but not all transcripts of the target gene, and 9% do not target any currently annotated transcript.

## Conclusion

Although alternative splicing is a ubiquitous and functionally critical phenomenon in eukaryotic gene expression, fluent software tools have not been available to assist researchers, particularly bench biologists, in determining which splice variants are targeted by particular qRT-PCR primer sets, RNAi effectors, microarray platforms or peptide-targeting reagents. Increasingly, all of those methods are being used, separately or in combination, to analyze gene function. SpliceCenter's integrated suite of applications for correlating experimental findings with the transcriptional structure of a gene should significantly aid in elucidating the roles played by splice variation in a wide range of biological processes and diseases. The SpliceCenter applications, currently including Primer-Check, Array-Check, siRNA-Check, and Peptide-Check, provide user-friendly, web-based tools for the biologist and bioinformaticist.

## Availability and requirements

The SpliceCenter website is available for use by academic, government, or commercial users without restriction or charge. The address of the site is: . We recommend using Internet Explorer (version 6.0 or better) or FireFox (version 2.0 or better) but are not aware of compatibility issues with other browsers.

## Authors' contributions

MCR and JAC designed and implemented the SpliceCenter applications, with input by BRZ and JNW. ABK designed and implemented the EVDB database and EVDB build process. MCR drafted the manuscript, with extensive input by NJC, BRZ, and JNW. NJC contributed to the design of the siRNA-Check application. All authors gave approval of the final version to be published.

## Supplementary Material

Additional File 1 This file contains the Microarray Database schema, and a use case comparison of a task performed either with SpliceMiner or with SpliceCenter. Click here for file

Additional File 2 siRNA-Check. (a) Graphical output from siRNA-Check for siRNAs designed to target *BAD*. Note that siBAD.2 does not target NM_032989. (b) Graphical output from siRNA-Check for siRNAs corresponding to the *YWHAZ *gene. Note that siYHWAZ.1 targets NM_003406 but not NM_145690. See Martin, *et al*.[3] for additional details. Click here for file

Additional File 3 Peptide-Check Graphical output from Peptide-Check for peptides designed to target isoforms of p53 Peptide 1: DQTSFQKENC – p53β, Peptide 2: MLLDLRWCYFLINSS – p53γ (See [21,25] for further details). Click here for file
