# Supplementary material for: SpliceCenter: A suite of web-based bioinformatic applications for evaluating the impact of alternative splicing on RT-PCR, RNAi, microarray, and peptide-based studies
Source: BMC Bioinformatics. 2008 Jul 18;9:313. doi: 10.1186/1471-2105-9-313 (PMC2491637; doi:10.1186/1471-2105-9-313)
Supplement: Additional File 1 — This file contains the Microarray Database schema, and a use case comparison of a task performed either with SpliceMiner or with SpliceCenter. [file 1471-2105-9-313-S1.doc]

**SpliceCenter: a suite of web-based bioinformatic applications for evaluating the impact of alternative splicing on microarray, RT-PCR, RNAi, and protein-based studies.**

**Ryan et al.**

**Supplementary information**

Microarray database schema

**Probe**

array

probe_id

probe_name

probe_type

symbol

degen_flag

**Probe_Sequence**

probe_id

sequence

**1**

**ProbeAddress**

probe_id

probe_chr_start

probe_chr_stop

**1**

**1..***

**Array**

organism_id

name

intname

groupname

seqnum

**1**

**1..***

**1**

**ProbeHit**

probe_id

acc

exon

probe_trans_start

probe_trans_stop

**1**

**1..***

Time savings of SpliceCenter utilities vs. our previous application, SpliceMiner

SpliceCenter provides “niche” applications focused on the splice variant targeting of a set of common microbiology technologies. The focused nature of the applications provides significant ease-of-use and time savings advantages when compared to our previous general purpose SpliceMiner utilities. To illustrate this point, we have provides a use case comparison of the time and steps required to perform a verification of RT-PCR primers in both tools. The purpose of the use case is to ensure that RT-PCR primers are targeting the same splice variants as a microarray probeset.

Task: Verify that RT-PCR primers and an Affymetrix U133 probeset are targeting the same splice variants.

**SpliceMiner (previous work)**

| Step | Description | Time (Minutes) |
| --- | --- | --- |
| 1 | Obtain probe sequences each probe in the probeset from the Affymetrix site. Either download the full sequence file and extract or use NetAFFX queries. (Time depends on familiarity with their site. Most other vendors have a way to get probe sequence but it is not always easy to find) | 15 - 60 |
| 2 | Obtain RT-PCR primer sequences. This is easy if the primers are custom. If not, use vendor materials to get primer sequence or reference sequence. | 5 |
| 3 | Format a batch FAST file with microarray probe and RT-PCR sequences. Label each primer/probe sequence uniquely. | 5 |
| 4 | Submit the fast query file to SpliceMiner’s batch sequence query page (interactive page only searches on one sequence at a time so it would be difficult to integrate all of the results) | 2 |
| 5 | Wait for batch results | 1 |
| 6 | Become familiar with SpliceMiner tabular output results. Manually build a list of the variants targeted by the probes (there will be one result line per probe per variant targeted). | 15 |
| 7 | Determine the variants targeted by **BOTH** the PCR primers from the tabular output. Note: SpliceMiner will only be able to match primers > 21 nts long – a new sequence matching engine was added to SpliceCenter for short sequence queries. | 5 |
| 8 | Using the results of steps 6 and 7, determine if the primers and probes are targeting the same variants. Use the interactive SpliceMiner gene symbol query to get a feel for the splice variant topology to understand the results | 5 |
|  | Total Time: | 53-98 minutes |

**SpliceCenter – same task**

| Step | Description | Time (Minutes) |
| --- | --- | --- |
| 1 | Obtain RT-PCR primer sequences. This is easy if the primers are custom. If not, use vendor materials to get primer sequence or reference sequence. | 5 |
| 2 | Use the interactive Primer-Check application. Enter primer sequences and select the check box for the Affemetrix U133A microarray | 1 |
| 3 | Look at graphical results to see which variants are targeted by the primers and which are targeted by the probset. Use the dropdown filter to select individual probesets if necessary. | 1 |
|  | Total Time: | 6 minutes |
