# Supplementary figures and images for: SpliceCenter: A suite of web-based bioinformatic applications for evaluating the impact of alternative splicing on RT-PCR, RNAi, microarray, and peptide-based studies
Source: BMC Bioinformatics. 2008 Jul 18;9:313. doi: 10.1186/1471-2105-9-313 (PMC2491637; doi:10.1186/1471-2105-9-313)

a

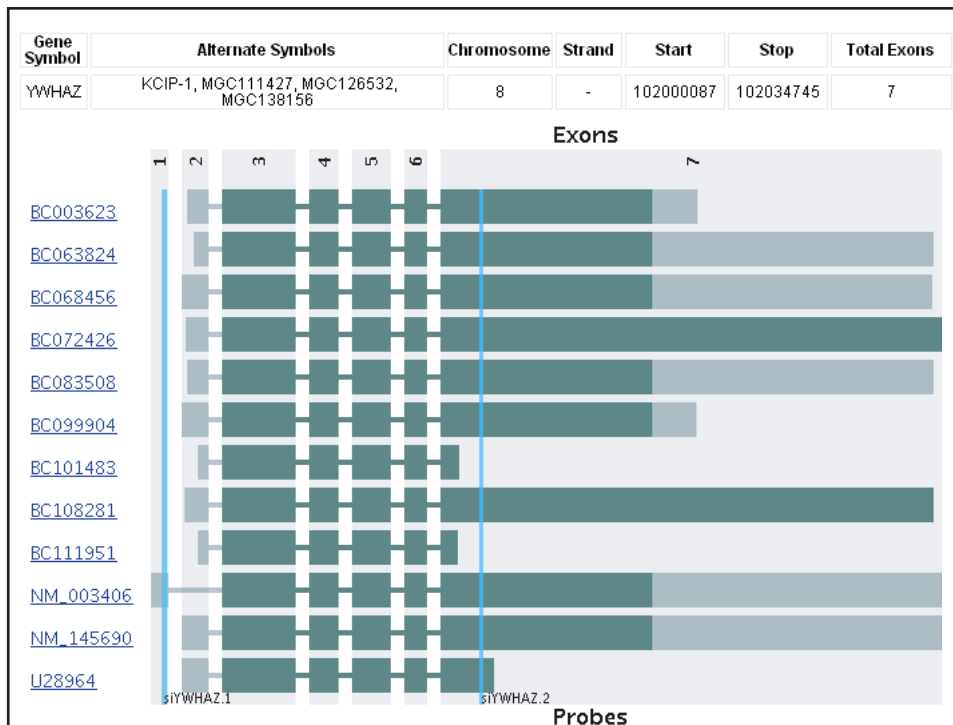

b

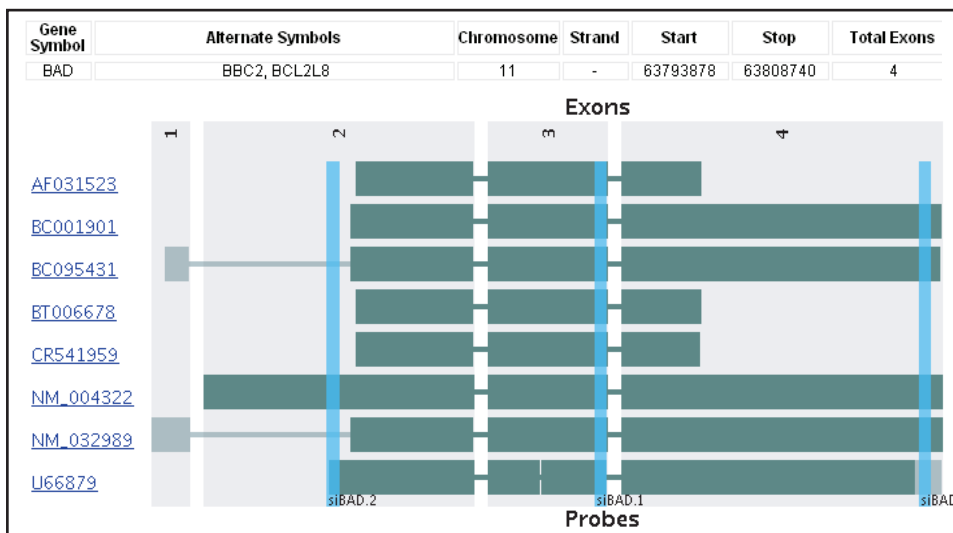

Supplement: Additional File 2 — siRNA-Check. (a) Graphical output from siRNA-Check for siRNAs designed to target BAD. Note that siBAD.2 does not target NM_032989. (b) Graphical output from siRNA-Check for siRNAs corresponding to the YWHAZ gene. Note that siYHWAZ.1 targets NM_003406 but not NM_145690. See Martin, et al.[3] for additional details. [file 1471-2105-9-313-S2.pdf]

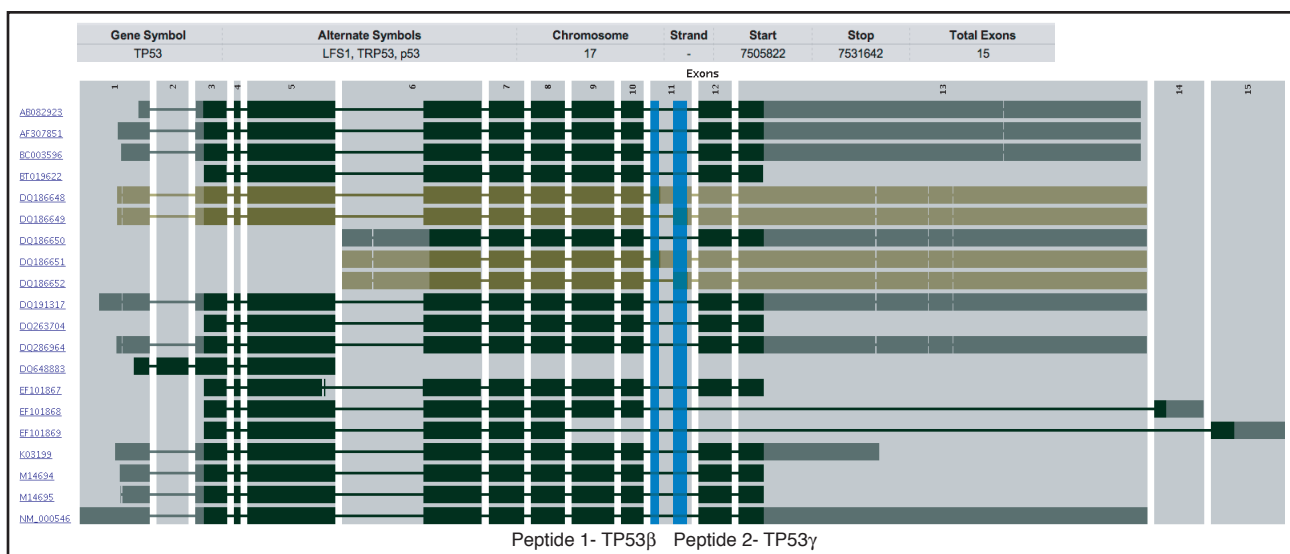

Supplement: Additional File 3 — Peptide-Check Graphical output from Peptide-Check for peptides designed to target isoforms of p53 Peptide 1: DQTSFQKENC – p53β, Peptide 2: MLLDLRWCYFLINSS – p53γ (See [21,25] for further details). [file 1471-2105-9-313-S3.pdf]
